# Isolation of the South China Sea from the North Pacific Subtropical Gyre since the latest Miocene due to formation of the Luzon Strait

**DOI:** 10.1038/s41598-020-79941-4

**Published:** 2021-01-15

**Authors:** Shaoru Yin, F. Javier Hernández-Molina, Lin Lin, Jiangxin Chen, Weifeng Ding, Jiabiao Li

**Affiliations:** 1 Key Laboratory of Submarine Geosciences, Second Institute of Oceanography, Ministry of Natural Resources, Hangzhou, 310012 China; 2 Department of Earth Sciences, Royal Holloway, University of London, Egham, TW20 0EX Surrey UK; 3 Guangzhou Marine Geological Survey, Guangzhou, 510075 China; 4 Key Laboratory of Gas Hydrate, Qingdao Institute of Marine Geology, Qingdao, 266071 China; 5 Laboratory for Marine Mineral Resources, Qingdao National Laboratory for Marine Science and Technology (Qingdao), Qingdao, 266071 China

**Keywords:** Ocean sciences, Solid Earth sciences

## Abstract

The North Pacific subtropical gyre (NPSG) plays a major role in present global ocean circulation. At times, the gyre has coursed through the South China Sea, but its role in the evolutionary development of that Sea remains uncertain. This work systematically describes a major shift in NPSG paleo-circulation evident from sedimentary features observed in seismic and bathymetric data. These data outline two contourite depositional systems—a buried one formed in the late Miocene, and a latest Miocene to present-day system. The two are divided by a prominent regional discontinuity that represents a major shift in paleo-circulation during the latest Miocene (~ 6.5 Ma). The shift coincides with the further restriction of the South China Sea with respect to the North Pacific due to the formation of the Luzon Strait as a consequence of further northwest movement of the Philippine Sea plate. Before that restriction, data indicate vigorous NPSG circulation in the South China Sea. Semi-closure, however, established a new oceanographic circulation regime in the latest Miocene. This work demonstrates the significant role of recent plate tectonics, gateway development, and marginal seas in the establishment of modern global ocean circulation.

## Introduction

In the Pacific Ocean, the North Pacific subtropical gyre (NPSG) represents a surface level water mass that advects warm water from the tropics to central and higher-latitude areas of the North Pacific basin. The water becomes cooler and then moves back toward the equator^1^. This anticyclonic gyre thus plays a major role in the export of energy, carbon and other nutrients to the deep ocean^2^.

**Figure 1:**
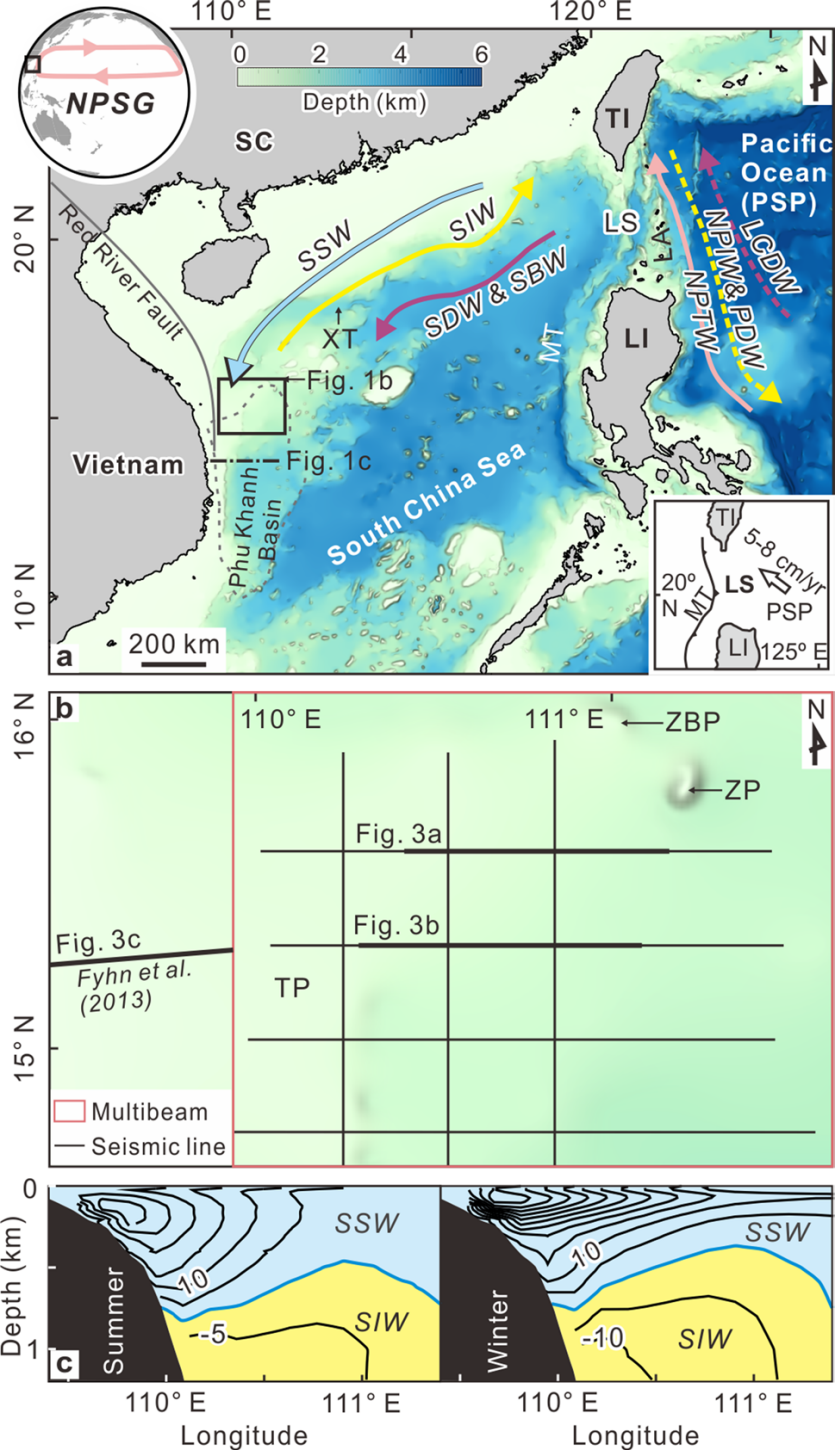
**(a)** Study area location (black rectangle) including water mass circulation. The black arrow marks the sense of movement of the plate. The gray line shows the location of Red River Fault. Dotted gray lines show the boundaries of Phu Khanh basin. Dotted-dashed line marks the hydrographic line in panel **(c)**; **(b)** data used in this study, including bathymetric survey and surface trace of seismic surveys; and **(c)** observations of 20-year average current velocity (cm/s) from 1992 to 2011 (data were provided by Huijie Xue), where positive velocity values denote southward flow and negative values denote northward flow. The blue line marks the zero contours. Contour interval is 5 m. *TI* Taiwan Island, *LI* Luzon Island, *PSP* Philippine Sea plate, *LS* Luzon Strait, *LA* Luzon arc, *SC* South China, *MT* Manila Trench, *XT* Xisha Trough, *TP* Triton platform, *ZP* Zhongjian platform, *ZJB* Zhongjianbei platform. Abbreviations for water masses given in text. Maps were generated using Surfer software (version 13, https://www.goldensoftware.com/products/surfer) and CorelDraw Graphics Suite X8 (https://www.coreldraw.com/cn/). Elevation data source: ETOPO1 (https://doi.org/10.7289/V5C8276M).

The NPSG does not presently enter the semi-closed South China Sea. The relatively recently formed Luzon Strait (Fig. 1)—an oceanic gateway comprising Luzon Island, Luzon volcanic arc and Taiwan Island—deflects the western side of the gyre^1^. During the middle to late Miocene, when Taiwan Island had not yet formed and Luzon Island had not moved northward to its present position^3–7^, the South China Sea was completely open to the Pacific Ocean. In that configuration, the NPSG or deeper water masses may have influenced heat transfer and carbon transport to and from the South China Sea. It might also have influenced sedimentary processes in the North Pacific Ocean.

This study used sedimentary and oceanographic evidence to determine the timing of isolation of the South China Sea from the NPSG. Ancient and modern sedimentary features related to the bottom current activity (contourite features) in the western South China Sea can help decode: (a) the timing of restriction and isolation of the basin, and (b) regional and global paleo-oceanographic implications.

## Regional setting

### Geological setting

The South China Sea was formed during the Oligocene to middle Miocene (33–15 Ma^8^), with subsequent eastward subduction of the South China Sea lithosphere along the Manila Trench due to northwestern (N307°) movement of the Philippine Sea plate at a rate of 5–8 cm/year^9–11^. Subduction was followed—in the middle to late Miocene—by further northward movement of Luzon Island and oblique (N307°) collision between the N–S trending Luzon volcanic arc of the Philippine Sea plate and the NE–SW trending northern South China margin of the Eurasian plate. Although the oblique collision initially occurred north of Taiwan, at 12–6.5 Ma, it gradually propagated southwestward to the present position^5,6^. Arc-continent collision resulted in uplift of Taiwan Island, an event referred to as the Taiwan orogeny^5,7^. As a consequence, the Luzon Strait formed, with Taiwan Island to the north and Luzon Island to the south.

**Figure 2:**
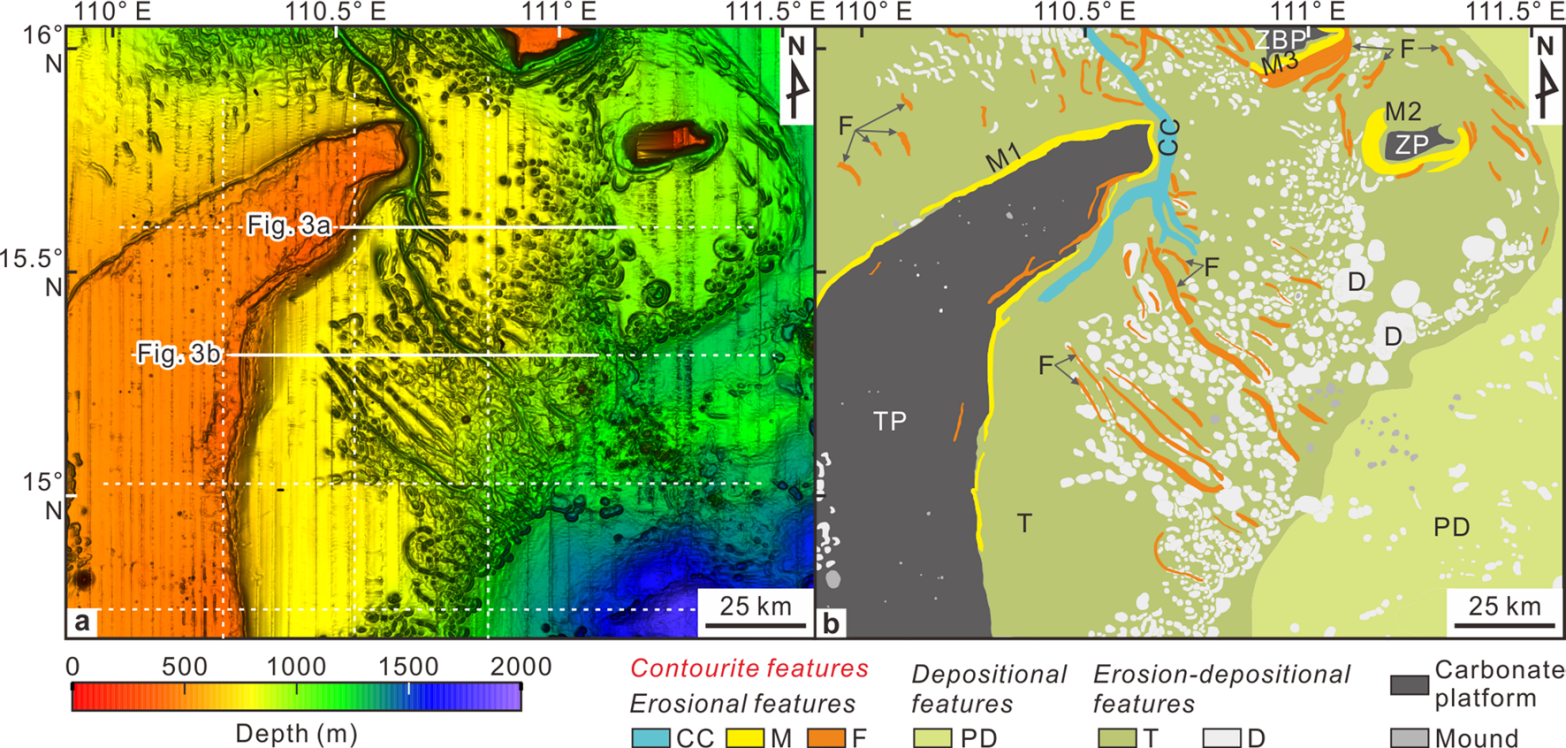
**(a)** Multibeam bathymetric map; and b) Morphosedimentary interpretation including large features. White lines in “a” show seismic lines position in this study. *CC* contourite channel, *M* moat, *F* furrow, *PD* plastered drift, *T* terrace, *D* depression. Maps were generated using Surfer software (version 13, https://www.goldensoftware.com/products/surfer) and CorelDraw Graphics Suite X8 (https://www.coreldraw.com/cn/).

Our study area includes the Central Vietnam slope in the western South China Sea (Fig. 1), which hosts three main carbonate platforms: the Triton (Guangle), Zhongjian and Zhongjianbei (Fig. 2). These platforms began to develop in the early Miocene on the Eocene–Oligocene basement highs, then became less active in the late Miocene due to regional thermal subsidence and cooling^12,13^. Main sediment input in the study area is from west and north^14,15^. Found in the Central Vietnam shelf is a slip-strike fault system, the Red River Fault (Fig. 1), which is dextral at a low rate after its reversal from sinistral during the late Miocene (e.g. Zhu et al., 2009^16^). Previous research has revealed contourite deposition (honeycomb structures) west of the Zhongjianbei platform^17^, and contourite channels around the Triton and Zhongjian platforms^18–20^.

### Oceanographic setting

The western South China Sea hosts four major water masses^21–23^ (Fig. 1) occupying surface, intermediate, deep and bottom levels in the water column. The South China Sea surface water (SSW) circulates cyclonically between 0 and 750 m (Fig. 1c) at a speed of up to 100 cm/s^22^, but sometimes descends to over 1000 m water depth with potential density (σ_θ_) between 21.0 and 26.8 kg/m^3^. The South China Sea intermediate water (SIW) circulates anticyclonically between 750 and 1500 m water depth (Fig. 1c) at a speed of 5–15 cm/s^22^ with potential density between 26.8 and 27.6 kg/m^3^. The South China Sea deep (SDW) and bottom (SBW) waters flow cyclonically below 1500 m with potential density of > 27.6 kg/m^3^. No direct current velocity observations of the SDW and SBW have been reported around the study area.

The northwest Pacific Ocean hosts four other major water masses^1^ (Fig. 1): North Pacific Tropical Water (NPTW), North Pacific Intermediate Water (NPIW), Pacific Deep Water (PDW) and Lower Circumpolar Deep Water (LCDW). The NPTW, driven by NPSG, circulates anticyclonically between 0 and 500 m water depth. The NPIW and PDW respectively flow southward between 500 and 1500 m water depth, and between 1500 and 4000 m water depth. The LCDW flows northward below 4000 m water depth. Water exchange between the Pacific Ocean and the South China Sea mainly occurs through the Luzon Strait (Fig. 1), where the NPTW and PDW flow into the South China Sea while the SIW flows out of the South China Sea. Luzon Strait transport is estimated at 3 to 6.5 Sv (where 1 Sv = 1 × 10^6^ m^3^/s) based on long-term observations (review in Cai et al., 2020^24^).

## Data and methods

This study relies on multibeam swath bathymetry and multichannel seismic reflection profiles.

### Multibeam swath bathymetric surveys

The bathymetric survey, which covered the most of the study area (Fig. 1b), was conducted using a Kongsberg EM122 multibeam system. The original multibeam sounding data were processed by means of CARIS HIPS and SIPS software (version 8.1.9). The final high-resolution seabed digital terrain model was built at a 100 m grid resolution using the swath angle surface method of CARIS HIPS and SIPS software (Fig. 2).

The bathymetric data served to identify modern contourite features together with seismic data following the morphological and seismic criteria defined by Fauguères et al. (1999)^25^, Rebesco and Stow (2001)^26^, Nielsen et al., (2008)^27^, Rebesco and Camerlenghi (2008)^28^, and Rebesco et al. (2014)^29^.

### Seismic reflection data

Eight multichannel 2D seismic reflection profiles (Fig. 1b) were used in this study. Seven of the eight profiles were collected by the Guangzhou Marine Geological Survey during 2013 and 2017, and the other one is adapted from Fyhn et al. (2013)^14^, which was acquired in 2003. The seismic data below the seabed were processed by means of a standard pre-stack time-migration procedure (see Supplementary Table S1 online). The major processing steps of the seismic sections above the seabed include direct wave attenuation, amplitude recovery, high-pass filtering, and constant velocity (seawater sound velocity) stack.

The seismic data were used to (a) execute a seismic stratigraphic analysis through a conceptual approach^30^, (b) identify large contourite features according to accepted criteria^29^, and (c) formulate an oceanographic interpretation of water mass structure following methods outlined in Holbrook et al. (2003)^31^. Age-control of seismic stratigraphic analysis is adopted from previous studies, especially Vu et al. (2017)^15^, with calibration of eight exploration wells in or near this study area.

## Seismic analysis

**Figure 3:**
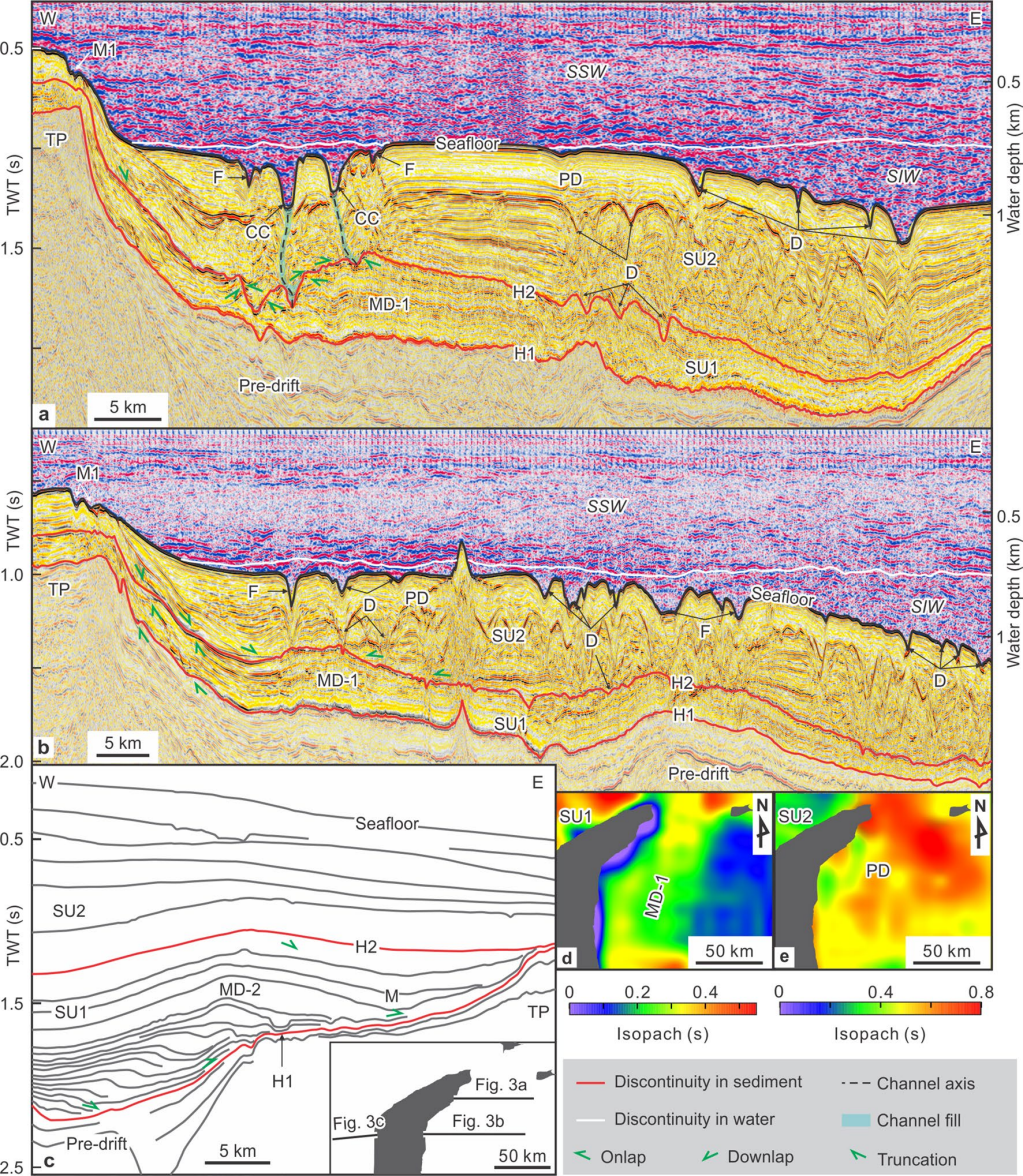
**(a,b)** Seismic profiles across the eastern flank of the Triton platform; **(c)** line drawings of a seismic profile across the western flank of the Triton platform (modified from Fyhn et al., 2013^14^); and **(d,e)** isopach maps for SU1 and SU2. Abbreviations for water masses, horizons, and seismic units given in text. Abbreviations for sedimentary features referenced to Fig. 2. Profile locations in Figs. 1, 2 and 3c. Seismic images in **(a,b)** were generated using GeoFrame software (version 4.5, http://slb-sis.com.cn/products-services/GeoFrame.aspx). The maps in **(d,e)** were generated using Surfer software (version 13, https://www.goldensoftware.com/products/surfer). All maps were designed using CorelDraw Graphics Suite X8 (https://www.coreldraw.com/cn/).

Within the water column, two major bodies can be discerned according to their reflectivity characteristics (Fig. 3). The first is a shallower body (between 0 and 750 m water depth) that appears in seismic data as high-amplitude reflections interbedded with low-amplitude reflections. This body corresponds to the vigorous South China Sea surface water (SSW). The second, deeper body (> 750 m water depth) exhibits moderate-amplitude reflections and corresponds to sluggish South China Sea intermediate water (SIW).

The sedimentary record shows three main seismic units (SUs) (Fig. 3). The lowermost SU represents a sedimentary package without contourite features (pre-drift). The two succeeding seismic units (SU1 and SU2, from bottom to top) consist mostly of large drift deposits. They are bound by two regional discontinuities (H1 and H2) and the modern seafloor. The H1 discontinuity defines the base of seismic unit SU1. This surface exhibits continuous, high-amplitude reflections and partial erosional truncation over the underlying pre-drift package around the Triton platform; the reflections of SU1 are of moderate amplitude, and moderate to continuous length (Fig. 3). Locally, the unit contains contorted, chaotic or free reflections below depressions and mounds.

The H2 discontinuity defines the base of seismic unit SU2. This surface is marked by continuous, high-amplitude reflections and partial erosional truncation over the underlying SU1 and around the east flank of the Triton platform. The SU2 reflections show onlap and downlap with respect to H2. The moderate-amplitude, relatively continuous reflections of SU2 are sometimes interrupted by discontinuous, contorted reflections within depressions.

## Contourite features

In both SU1 and SU2, a number of large erosional, depositional and erosion-depositional contourite features appear along the western South China Sea margin (Figs. 2 and 3).

### Erosional features

Three types of erosional features were identified.

#### (a) Contourite channels

A single contourite channel (larger valley) extends southward along the upper slope. This channel splits into four distributaries after traversing the passage between the Triton and Zhongjian platforms. It occupies a 700 to 900 m water depth, has a total length of 63 km, a width of 0.5–2.1 km, and incises to 130–220 m depth (Table 1). The channel starts at the H1 discontinuity and records cycles of erosion and infill within SU1.

**Table 1:** Morphological parameters and interpretation of contourite features in this study.

Contourite features			Length (km)	Width (km)	Depth/thickness (m)	Dip (º)	Azimuth (º)	Formation interpretation
Erosional	Channel		> = 63	0.5–2.1	130–220	0.2	46, 335	Cores of SSW currents
	Moat		30–100	0.3–3.1	20–140	< 0.2	0–202, 336–360	Cores of bottom currents
	Furrow		4–40	0.5–1.5	30–120	< 0.3	40, 62, 311,337, 351	Branches/filaments of bottom currents
Depositional	Plastered drift		> 170	< = 85	425–680	0.3–0.6	26, 334	SIW
	Mounded drift	MD-1	100	15–26	300–500	0.7–0.8	23	NPSG
		MD-2	–	> = 27	990	1.1	–	NPSG
Mixed	Terrace		> 170	51–69	323–510	0.2	26, 334	Erosion by SSW; deposition by SIW
	Depression		0.7–6.3	0.3–4.2	30–150	–	41, 312	Eddies/fluid flow escape

#### (b) Moats

These smaller valleys are associated with separate drifts identified in SU1 and SU2 along the carbonate platforms (Triton, Zhongjian and Zhongjianbei). The modern moats (M1, M2 and M3) occur at 450–1000 m water depth, extend 30–100 km in length and 0.3–3.1 km in width, and reach incision depths of 20–90 m (Table 1). In SU1, they extend along the western flank of the Triton platform (Fig. 3c) and migrate towards the east, where they reach 1.6–13.1 km in width and 50–140 m in depth.

#### (c) Furrows

These smaller erosional valleys run generally oblique to isobaths. At least 70 furrows could be identified between 450 and 1000 m water depth on the upper slope. They are 4–40 km in length, 0.5–1.5 km in width, and reach depths of 30–120 m (Table 1).

### Depositional features

Two types of sediment drifts were identified (Figs. 2 and 3).

#### (a) Plastered drifts

A plastered drift exhibits a broad, very slightly mounded geometry and thins both onshore and offshore within SU2 (Fig. 3). This feature extends along the upper slope between 600 and 1100 m water depth to cover the eastern and northern flank of the Triton platform. The drift is 170 km long, up to 85 km wide, and reaches 425–680 m in sedimentary thickness (Table 1). It exhibits an aggradational configuration, and is incised by the contourite channel and furrows described above.

#### (b) Mounded drifts

Two mounded drifts (MD-1 and MD-2) were identified in SU1 mainly on the basis of their external shape (Fig. 3). The MD-1 drift, 300–500 m thick and 15–26 km wide (Table 1), extends along the eastern flank of the Triton platform between 1.3 and 2.2 s (two-way travel time; twt). Whereas MD-1 exhibits continuous reflections with an aggradational configuration, the MD-2 drift is up to 990 m thick and at least 27 km wide, running along the western flank of the Triton platform between 1.2 and 2.2 s (twt). This feature has an aggradational and progradational (eastward) configuration. Moats are found along the eastern side of the drift.

### Erosion-depositional features

Two types of erosion-depositional (mixed) features were identified (Figs. 2 and 3).

#### (a) Contourite terraces

A contourite terrace appears along the upper slope at 600–1100 m water depth, exhibiting both erosional (contourite channel and furrows) and depositional (plastered drift) phases. It has a mean seafloor gradient of 0.2° (Table 1). This terrace represents the proximal sector of a plastered drift located along the middle slope.

#### (b) Depressions

Several thousand depressions commonly appear in groups. These features, dominated by sub-circular shape in plan view, are on average 0.7–6.3 km long, 0.3–4.2 km wide and 30–150 m deep (Table 1). Depressions are filled by contorted and/or sub-parallel reflections, and appear in SU2.

## Decoding circulation from the late Miocene to the present

In the present-day seafloor, the contourite terrace is basically located at an interface depth range between the modern SSW and SIW, which define the upper and lower sections of the water column (Fig. 3a, b). In winter, the interface of the two shifts to a shallower position relative to that of summertime (Fig. 1c). Above the interface the SSW circulates faster (up to 100 cm/s) than the SIW (5–15 cm/s^22^).

**Figure 4:**
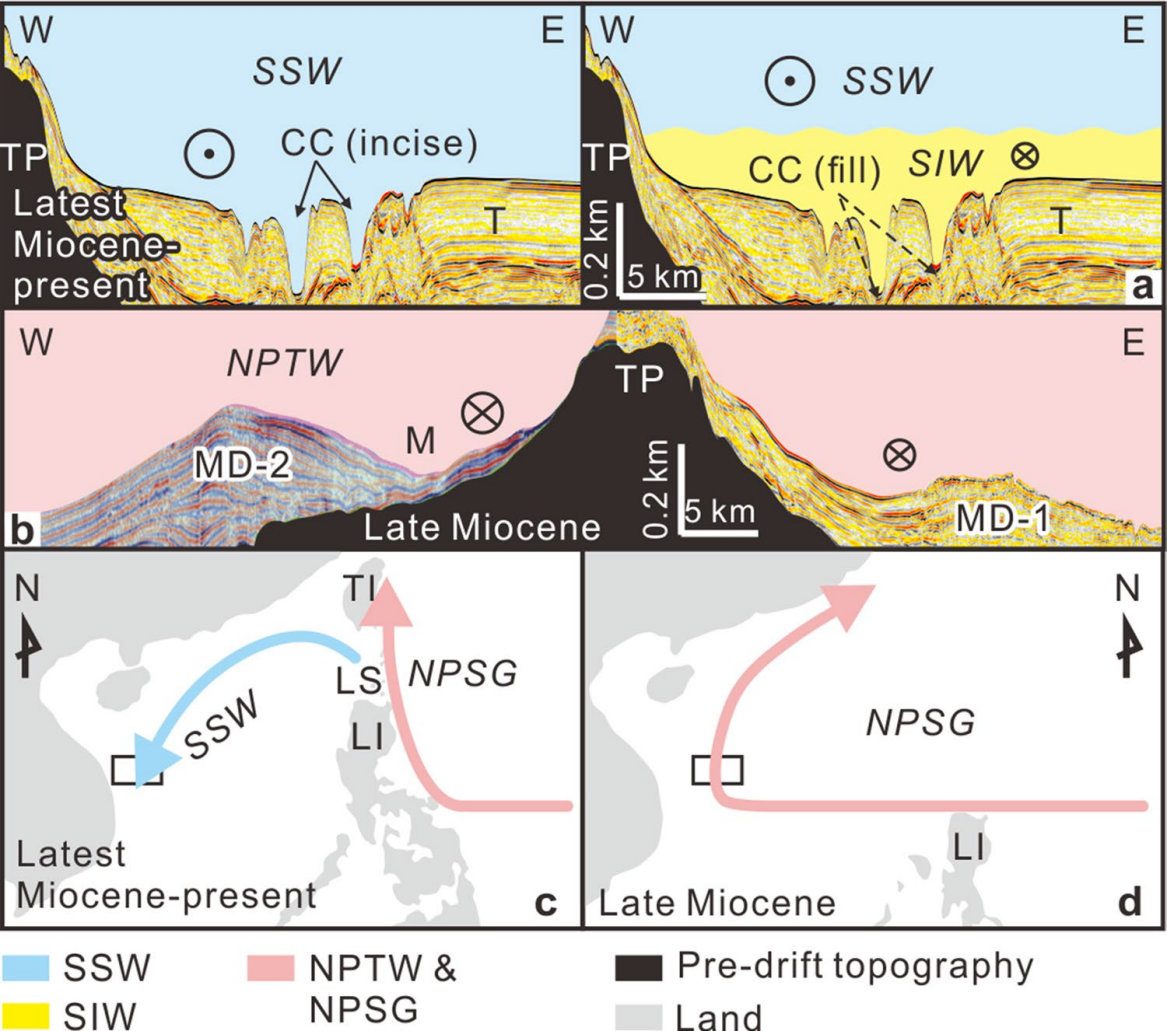
Sketches for the terrace and the contourite depositional systems for SU2 **(a)** and SU1 **(b)** formation under South China Sea circulation. Reconstruction of circulation from latest Miocene to present **(c)** and in late Miocene **(d)**. In panels **(a,b)**, current direction is indicated by the symbols:  circled times  = Northward flow and circled dot = Southward flow. Topography data source in **(c)** is ETOPO1 (https://doi.org/10.7289/V5C8276M, https://ngdc.noaa.gov/mgg/global/global.html). Topography in **(d)** is modified from Hall, 2012^4^. Abbreviations for water masses given in text. Abbreviations for sedimentary features referenced to Fig. 2. Seismic images in **(a,b)** were generated using GeoFrame software (version 4.5, http://slb-sis.com.cn/products-services/GeoFrame.aspx). All maps were designed using CorelDraw Graphics Suite X8 (https://www.coreldraw.com/cn/).

The terrace´s erosional features (contourite channel and furrows) record sweeping of the seafloor by cores, branches and filaments of faster currents within the water mass. A plastered drift—muddy, as inferred from exploring wells^15^—appears in areas with slower currents. Terrace formation thus implies vertical fluctuations of that interface in time, evoking the mechanism proposed in previous studies of terraces^32,33^ and plastered drifts^34^ in other marine basins. When the interface is deeper (i.e., below terrace depth), the terrace is eroded by the fast-flowing SSW (Fig. 4a); when the interface is shallower, the terrace comes under the weaker influence of the SIW (Fig. 4a), which favors depositional processes.

The oblique intersection of the furrows on isobaths along the terrace (Fig. 2) indicates the definite central effect of water mass circulation, although other processes beside water mass circulation—e.g. turbidity currents, internal waves, and cascading dense water—possibly make a contribution meriting further study. In addition, the furrows usually cross trains of depressions (Fig. 2), indicating the contribution of depression to the development of the furrows.

Based on the criteria of Faugères et al. (1999)^25^, the present position of the contourite channel with respect to the adjacent drift along the eastern flank of the Triton platform would indicate a southward-flowing SSW. Current data confirm the hypothesized Coriolis-induced imbalance in the geostrophic flow along the eastern side of the platform^22^. Both the contourite channel and the plastered drift appear within the present-day seafloor, but also throughout SU2 (Fig. 3). We can therefore infer that present-day SSW circulation was already established by the time discontinuity H2 occurred at the base of SU2. These features represent the onset of the modern contourite depositional system. The H2 boundary corresponds to the base of Sequence 5 from Vu et al. (2017)^15^, who dated eight wells as upper Upper-Miocene based on biostratigraphy (see Supplementary Table S2 and Fig. S1 online), to the base of Pliocene from Fyhn et al. (2013)^14^, and to T30 (base of Pliocene) from Lu et al. (2017, 2018)^35,36^. Growth of the muddy plastered drift during SU2 indicates the influence of a weak water mass (SIW) at this depth^25,34^. The seismic reflection variations in SU2 (Fig. 3) may be related to changes of the sluggish SIW intensity variation, sediment source change and other sedimentary processes.

A more deeply buried contourite depositional system appears within SU1, which is muddy, inferred from drilling results^15^. Contourite depositional features include mounded drifts MD-1 and MD-2, at a similar depth. They develop under conditions of enhanced bottom currents^25,29,34^. Interestingly, the distribution of the paleo-moat and the eastward progradation of buried mounded drift MD-2 along the western side of the Triton platform (Figs. 3c and 4b) would indicate a northward-flowing, Coriolis-induced geostrophic current concentrated along this side. Good correlations point to a late Miocene age for SU1, as its base (H1) correlates with the base of Sequence 4 from Vu et al. (2017)^15^ and T40 from Lu et al. (2018)^35^—both dated as the base of the late Miocene—as well as the base of late Miocene from Fyhn et al. (2013)^14^ (see Supplementary Table S2 and Fig. S1 online).

Assuming an average interval velocity of 1700 m/s for SU1 and SU2 deposition, the buried late Miocene contourite depositional system in SU1 lies 1175 to 1650 m below the modern sea surface level. The estimated paleo-water depth of the seabed was 20–50 m at the beginning of the late Miocene and about 500–700 m by the end of the late Miocene^37^, the deepening over time being a result of rapid post-rift subsidence in the late Miocene^12,37^. A buried contourite depositional system therefore formed under the influence of a surface water mass flowing northward along the western South China Sea (Fig. 4b).

The modern East Asian summer monsoon could have facilitated clockwise surface water circulation south of the study area, but not reaching it—the modern SSW in the study area is always southward^22,38^. During the late Miocene, the intensity of the East Asian summer monsoon would have been slightly weaker than in the Pliocene to present^39,40^. In other words, the late Miocene East Asian summer monsoon could not have driven the northward circulation of the SSW in the study area. Yet throughout the late Miocene, the South China Sea had a greater degree of exchange with the Pacific Ocean than today, owing to the much more southern position of Luzon Island and the non-appearance of Taiwan Island^7,9^. Consequently, the surficial anticyclonic North Pacific Tropical Water (NPTW) could connect directly with the South China Sea surface water (SSW), while the intermediate North Pacific Intermediate Water (NPIW) would have flown into the western South China Sea at the depth occupied by the present-day South China Sea intermediate water (SIW). Therefore, during the late Miocene deposition of SU1, a relatively vigorous water mass flowed north in a direction opposite that of nowadays. The direct input of the North Pacific subtropical gyre (NPSG) west of the South China Sea generated a different paleo-oceanographic setting.

## Isolation of the South China Sea from the North Pacific Subtropical Gyre

Buried and modern contourite depositional systems generated by water masses flowing in opposite directions provide evidence of major paleo-oceanographic shifts in the latest Miocene (Fig. 4c,d). Such changes indicate isolation of the South China Sea from the NPSG due to formation of the Luzon Strait as a consequence of the further northwest (N307°) movement of the Philippine Sea plate since the latest Miocene (~ 6.5 Ma)^5,7^. This event entails northward movement of Luzon Island, rapid uplift of Taiwan Island, and remarkable volcanism within the Luzon arc^7^—features forming a barrier for the westward extension of the NPSG and isolating the South China Sea. Deflection by the barrier caused a significant eastward shift in Pacific surface circulation^41^.

The isolation of the South China Sea exemplifies that restriction of a small marginal sea by plate tectonics can influence both regional circulation in marginal seas and open ocean circulation. It is noteworthy that all the small-scale depressions occur in SU2, most likely formed by erosion and deposition involving sub-mesoscale currents triggered by mesoscale eddies^17,42^, although fluid flow activity may also have contributed to the depression formation^35^. Accordingly, isolation might have significantly enhanced the activity of mesoscale eddies in the sea. Such oceanographic shifts can occur prior to the complete closure of gateways. A similar scenario affected the Sea of Japan, another semi-enclosed marginal sea isolated from the North Pacific during the late Miocene to middle Pliocene^43^.

The isolation and further restriction of the South China Sea roughly coincide with (at least) three major paleo-oceanographic events linked to global ocean circulation. First, the Central American Seaway became shallow enough to block intermediate water circulation between the Atlantic and Pacific Oceans^44^. Second, the narrowing of the Indonesian Seaway reduced equatorial water mass exchange between the Indian and Pacific Oceans^45^. Third, the Mediterranean/Atlantic connection closed, and then opened (Strait of Gibraltar) by the beginning of the Pliocene^46,47^. All these global changes, within the same time frame, contributed to the development of modern global ocean circulation.

## Conclusion

The South China Sea has been isolated from the NPSG since the latest Miocene due to formation of the Luzon Strait, in turn related to further northwest movement of the Philippine Sea plate. Isolation because of recent plate tectonic activity (including the closure and opening of other gateways) caused major coeval paleo-oceanographic and sedimentary changes within the South China Sea. Finally, the latest Miocene paleo-oceanographic change promoted the present-day circulation in both the Pacific Ocean and the South China Sea.

## Supplementary Information

Supplementary Table S1.

Supplementary Table S2.

Supplementary Figure S1.
