# Supplementary material for: Isolation of the South China Sea from the North Pacific Subtropical Gyre since the latest Miocene due to formation of the Luzon Strait
Source: Sci Rep. 2021 Jan 15;11:1562. doi: 10.1038/s41598-020-79941-4 (PMC7810843; doi:10.1038/s41598-020-79941-4)

Isolation of the South China Sea from the North Pacific Subtropical Gyre since the latest Miocene due to formation of the Luzon Strait

Shaoru Yin, F. Javier Hernández-Molina, Lin Lin, Jiangxin Chen, Weifeng Ding, Jiabiao Li

**Supplementary Figure 1** Seismic-well correlation to determine age of seismic discontinuities. (a) Location and names of three exploring wells closest to the study area, as well as location of the seismic profiles across the wells. Seismic-well correlation at wells CS-1X (b), CH-1X (c) and CVX-1X (d). Wells and associated seismic profiles are modified from Vu et al., 2017. S4, S5, S6 show the seismic sequences from Vu et al., 2017, which are related to this study. SU1 in this study corresponds to S4 from Vu et al., 2017. SU2 in this study corresponds to S5 and S6 from Vu et al., 2017. Maps were generated using Surfer software (version 13, <https://www.goldensoftware.com/products/surfer>) and CorelDraw Graphics Suite X8 (https://www.coreldraw.com/cn/).


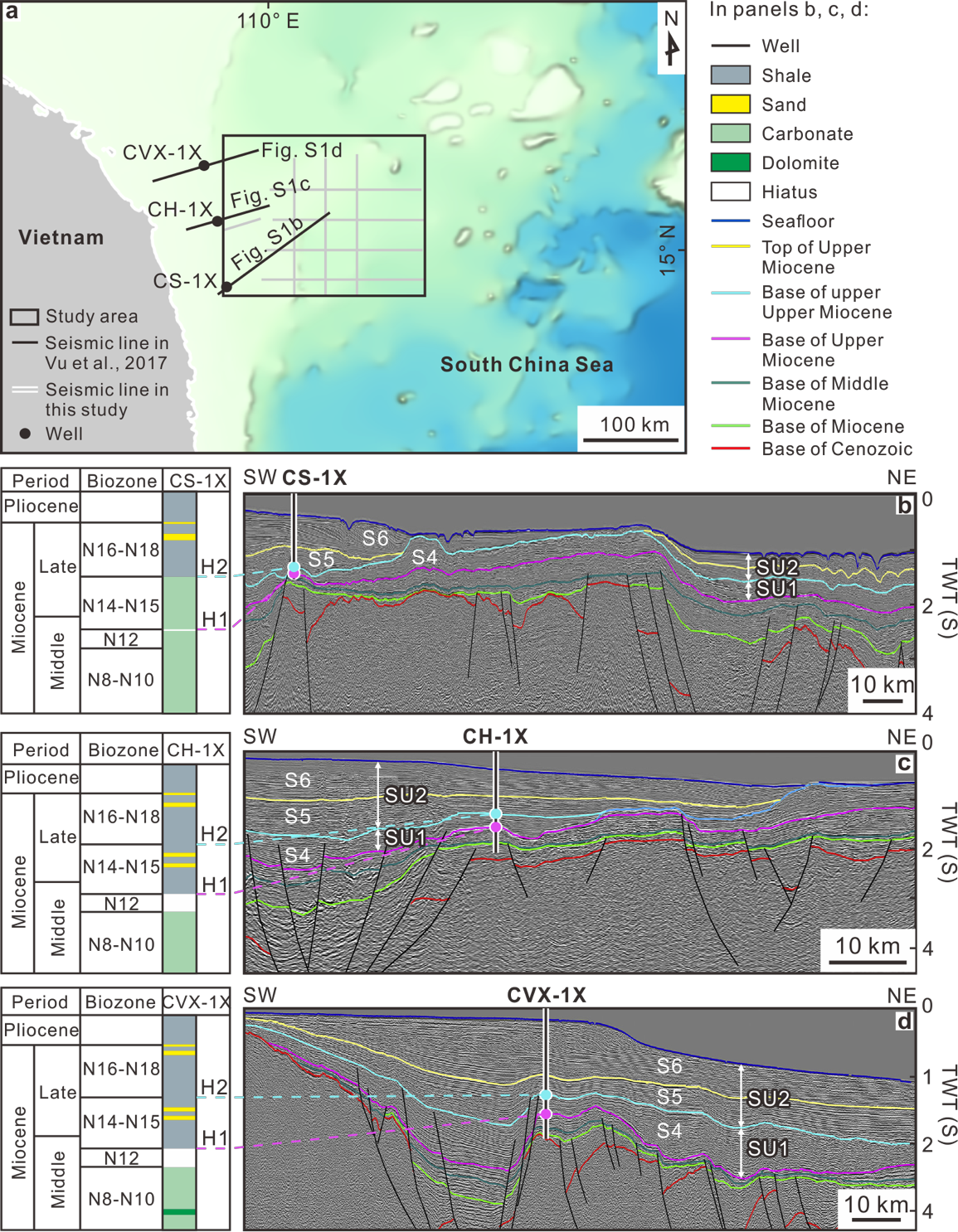

Supplement: Supplementary file 3 — Supplementary Figure S1. [file 41598_2020_79941_MOESM3_ESM.docx]
